# Supplementary figures and images for: Access Site Bleeding Complications with NOACs versus VKAs in Patients with Atrial Fibrillation Undergoing Cardiac Implantable Device Intervention
Source: J Clin Med. 2022 Feb 14;11(4):986. doi: 10.3390/jcm11040986 (PMC8876635; doi:10.3390/jcm11040986)

Figure S1

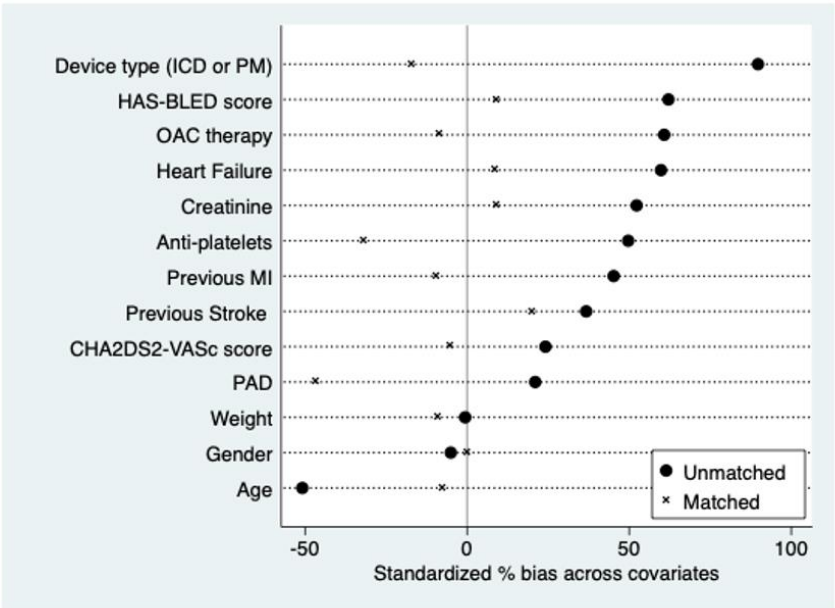

Figure S2

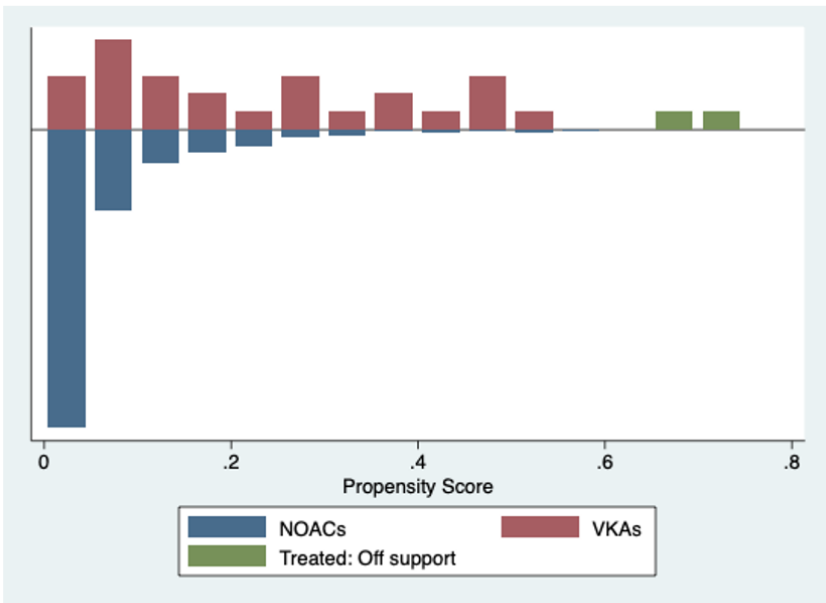

Supplement: Supplementary file 1 [file jcm-11-00986-s001.zip › jcm-1597751-SI.pdf]
